# Clinical diagnosis and histological analysis of vocal nodules and polyps

**DOI:** 10.5935/1808-8694.20130078

**Published:** 2015-10-08

**Authors:** Raquel Buzelin Nunes, Mara Behlau, Mauricio Buzelin Nunes, Juliana Gomes Paulino

**Affiliations:** a Speech and Hearing Therapist, MSc in Speech Therapy - Pontifical Catholic University of São Paulo, Specialist in Voice - Center of Voice Studies (Owner of Fonolife Speech Clinic).; b Speech and Hearing Therapist. PhD in Human Communication Disorders, São Paulo State University (Director of the Center of Voice Studies).; c MD. Pathologist. Specialist in Anatomic Pathology by the SBP. Specialist Cytopathologist - SBC. (Pathologist at Santa Casa/Owner and Technical Director - Moacyr Junqueira Institute).; d MD. Otorhinolaryngologist - Instituto de Otorrinolaringologia de MG.

**Keywords:** histology, otolaryngology, voice

## Abstract

Recent studies emphasize the importance of the clinical-histology correlation in laryngeal pathologies.

**Objective:**

To compare the ENT diagnosis with the pathology diagnosis one of 132 surgical specimens, from 119 patients with vocal nodules and polyps.

**Method:**

Retrospective study. We investigated the paraffin blocks corresponding to the lesions of the operated patients. We made new histology cross-sections, totaling 396 new slides, divided into three groups: hematoxylin and eosin, Gomori trichrome and PAS. We analyzed the following histological parameters: epithelium, *lamina propria*, basement membrane, vascular changes. We compared the laryngological and pathological diagnoses, and we did the statistical analysis, checking the predominant histological aspects in each lesion.

**Results:**

There was an agreement between the clinical and pathological diagnoses in 123 (93.18%) of 132 lesions analyzed (42.42% nodules and 50.76% polyps). In the histological parameters we found: epithelial changes such as nodules hyperplasia (82.14%) and polyp atrophy (31.34%). *Lamina propria*: edema in polyps (71.43%), fibrosis in the nodules (57.14%). Basement membrane: thickened nodules (100%), thin/no change in polyps (100%). There was a predominance of vascular changes in the polyps.

**Conclusion:**

We found a high correlation between the ENT diagnosis and the pathology report. Histopathologically, the nodules presented with predominantly epithelial changes, *lamina propria* and basement membrane fibrosis, while the polyps by changes strictly on the *lamina propria* and vascular aspects.

## INTRODUCTION

In recent decades, the development of new noninvasive diagnostic methods and advances in the study of semiology, laryngeal physiology and histopathology are allowing a thorough assessment of phonation, especially the interference of laryngeal lesions in the layers of the vocal folds.

Among the most common benign laryngeal lesions treated in specialized voice clinics are vocal nodules and polyps, which diagnosis is made primarily by patient history, clinical complaints and through visual examination such as indirect laryngoscopy with rigid or flexible fiber optic scope and stroboscopy. Its etiology is related to vocal abuse. Nodules are usually formed from constant vocal abuse over time, while polyps may originate from a single episode of abuse. Nodules have good results in speech therapy; and polyps are more resistant and there are some recent reports in the literature of these lesions being resorbed with speech therapy without surgery^1^, ^2^, ^3^.

It is clear, therefore, the great relevance of ENT diagnosis. It is not always that the otolaryngologist can establish the ultimate diagnosis through clinical manifestations alone, thus resorting to surgery and the pathology diagnosis, which check the histological characteristics of the lesions to confirm the type of disorder. Often, these diagnoses are not concordant

This disagreement may be justified by the fact that these pathologies are theoretically different. While for otolaryngologists these lesions are well differentiated, pathologists do not see this differentiation. Microscopically, nodules and polyps are defined as identical lesions resulting from phonotrauma with or without stress, inflammatory irritation and allergic factors that develop mainly in the anterior third of the vocal folds causing hoarseness. The microscopic appearance depends on the stage of the lesion (whether it is called a polyp or a nodule): in the beginning there is edema, fibroblast proliferation and later, vascular and stromal hyalinization. Many histological features suggest vascular or hemorrhagic origin^4^.

Studies on the histological architecture of the vocal folds have been modifying procedures both in laryngeal surgery, as well as in the forms of speech therapy. A clinical-histological correlation is not always easy, but an accurate diagnosis is of the utmost importance.

The differences found in histopathological studies led us to realize the need to better understand the action of these lesions in the cellular matrix and on the vibration of the vocal folds, and in the future it may assist in the development of new therapeutic techniques.

The aim of this study is to compare the ENT clinical diagnosis with the pathology reports of vocal fold nodules and polyps from the surgical specimens of 119 individuals and investigate the histological features that differentiate these lesions.

## METHOD

Since we worked with archive material, this study was deemed riskless and approved by the ethics committee of the institution, under number 0125/2002. This is a retrospective cross-sectional study.

For this study we selected 132 lesions diagnosed as vocal fold nodules and polyps, yielding a total of 57 nodules and 75 polyps from 119 patients of both genders, aged 18-60 years, submitted to laryngeal microsurgery between the years 1999 and 2002. The clinical diagnosis of the lesions was performed by only one Laryngologist, through videolaryngo-stroboscopy and laryngeal microsurgery.

The otorhinolaryngologist considered the following characteristics for the clinical diagnosis: vocal nodules characterized as rounded, sessile and whitish lesions, located in the anterior or middle thirds of the vocal folds in the membranous part, symmetrical in size and location, bilateral, associated with a mid posterior cleft or double cleft. Polyps are characterized as unilateral lesions, sessile or pedicled, located in the anterior and middle thirds of the vocal folds.

The paraffin blocks of these lesions were collected from the pathology laboratory archives. Subsequently, new 6-micron slices were made and the material was placed on glass slides. These cross-sections were stained by hematoxylin and eosin (HE), periodic Schiff acid (PAS) and Masson's trichrome for a total of 396 new slides. The slides were further divided into three groups of 132 slides, in accordance with the kind of color used for microscopic analysis. For histological characterization of the lesions we drafted an analysis protocol with the following parameters: epithelium, *lamina propria*, basement membrane and vascular alterations. These parameters were defined according to the histological characteristics of laryngeal nodules and polyps that could be observed histologically from the type of stain used.

For the epithelium parameter we selected the following alterations: hyperplasia, atrophy, erosion, dysplasia and keratinization such as parakeratosis and/or orthokeratosis. Hyperplasia was defined as an increase in cell number and epithelium thickness caused by stimulation or trauma; atrophy as a reduction in the number of cells and epithelium thinning, erosion as shallow ulceration of the epithelium without reaching the *lamina propria*, dysplasia as epithelial disorganization with cellular atypia and keratinization as the layer formed by cells that slough off the surface epithelium - it may be complete (orthokeratosis) or incomplete (parakeratosis).

For the *lamina propria* parameter we considered changes such as: edema, inflammatory infiltrate and fibrosis. Edema was characterized by fluid leakage into the interstitial space; fibrosis by increasing the connective stroma arising from normal or excessive healing and inflammatory infiltration by inflammatory cell exudation.

The basement membrane is a laminate structure located between the epithelium and the *lamina propria* surface layer. To analyze this parameter we selected the following aspects: diffuse thickening - when most of the epithelium was thickened, focal thickening - when some parts of the epithelium were found thickened; and thinned (fine) or without change: when there was no thickening in any part of the basement membrane.

Finally, in the vascular changes parameter we analyzed the presence of amorphous material deposit (AMD), light angiectasia, vascular clusters with marked angiectasia, hemosiderin and recent hemorrhage. AMD was defined as material without a defined structure; mild angiectasia, such as small vessels; vascular clusters with marked angiectasia, as large vessels; recent hemorrhage as blood output from the vascular bed, and hemosiderin, as pigment deposits containing iron caused by the degradation of overflowing erythrocytes.

To check for epithelial changes: edema, inflammatory infiltrate, and vascular alterations, we used HE staining. For fibrosis we used the Gomori trichrome and PAS to assess the basement membrane because they better evidence these aspects.

The slides were analyzed by a pathologist and the speech therapist through a light microscope coupled to a 14-inch TV, without prior knowledge concerning the ENT diagnosis. The features found were described in a consensus between the pathologist and the speech therapist, as present or absent for each parameter analyzed. The final histological diagnosis of the type of lesion was performed by the pathologist.

The pathological diagnosis was compared with the ENT diagnosis by the speech therapist and classified as concordant or discordant.

The data was analyzed using the Chi-square statistical test and the Fisher's exact test when the Chi-square test was not possible. Statistically significant differences were considered when the *p*-value was less than 0.005. The analyses were performed through the 13.0 Inc and S-Plus version 2000 software packages.

## RESULTS

Regarding the frequency of nodules and polyps in relation to gender, there was a prevalence of vocal nodules in females (86%) and polyps in males (64%). There were differences concerning the ENT and pathology diagnoses of the lesions. The otolaryngologist diagnosed 57 nodules and 75 polyps; and the pathologist, 64 nodes and 68 polyps. (Tables 1 and 2).

**Table 1:** Number and percentage distributions of polyps and nodules frequency in relation to gender.

Genders						
Lesion	Males		Females		Total	
	N	%	N	%	N	%

Nodules	8	14.03	49	85.99	57	100
Polyps	48	64.00	27	36.00	75	100

**Table 2:** Number and percentage distribution of ENT and pathology diagnoses of vocal fold nodules and polyps.

Diagnosis	Nodule		Polyp		Total	
	N	%	N	%	N	%
Otorhinolaryngological	57	43.18	75	56.82	132	100
Pathological	64	48.48	68	51.52	132	100

In comparing the clinical and histological findings, there was agreement in 123 (93.18%) lesions from the 132 lesions analyzed, making up a total of 56 nodules (42.42%) and 67 polyps (50.76%). Only nine (6.82%) lesions had different diagnoses (Table 3).

**Table 3:** Comparison between the ENT and the pathology diagnoses of vocal fold nodules and polyps.

	Nodule		Polyp	
	N	%	N	%
Pathology report = Otolaryngological	56	42.42	67	50.76
Pathology report ≠ Otolaryngological	1	0.76	8	6.06

**Figure 1:**
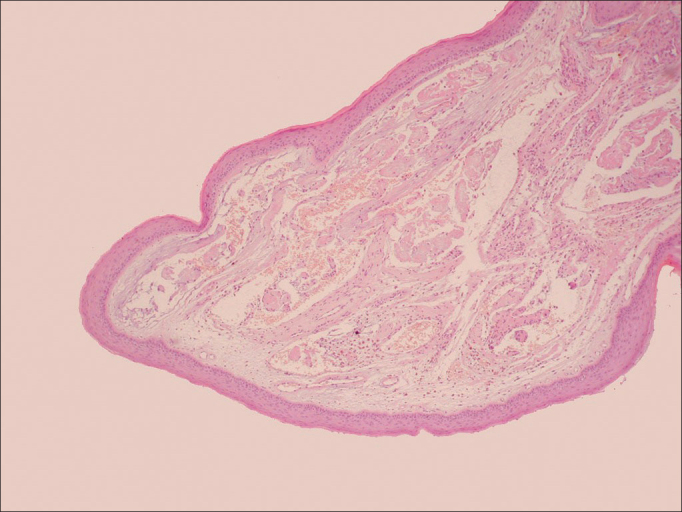
Histology of Vocal Cord Polyp. Overview of the vocal fold polyp histology stained with hematoxylin and eosin (HE 20x). Notice the intact epithelium and the thin basement membrane. In the lamina propria there are vascular changes, amorphous material deposits, past hemorrhage and hemosiderin.

**Figure 2:**
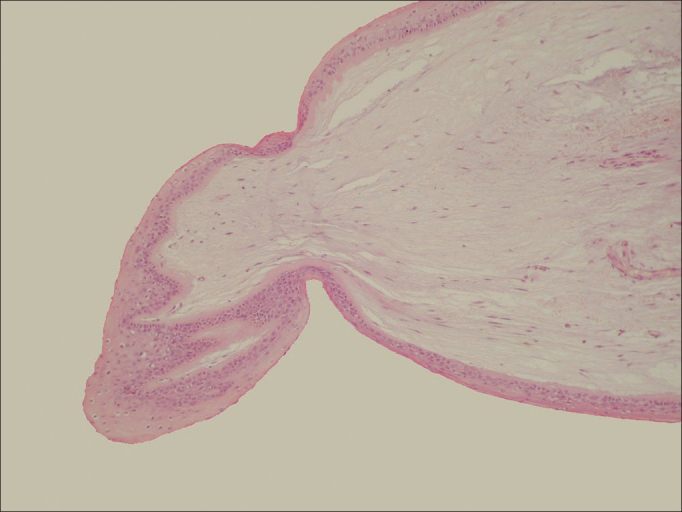
Histology of the Vocal Cord Nodule. Overview of the vocal fold nodule histology stained with hematoxylin and eosin (HE 20x). Presence of hyperplastic epithelium, basement membrane thickening and few changes in the lamina propria.

The analysis of histological parameters yielded predominantly epithelial type hyperplasia nodules (82.14%) and atrophy in polyps (31.34%). The keratinization type of parakeratosis in nodules (33.93%). In the *lamina propria*, 71.43% of the polyps had edema and 57.14% of nodules had fibrosis. The basement membrane was thickened in nodules (100%) and thin/intact in polyps (100%). As for the presence of vascular changes, mild angiectasia predominated in nodules (80.36%), while other aspects predominated in polyps: deposit of amorphous material (73.13%), presence of vascular clusters with marked angiectasia (76.12%), recent hemorrhage (76.12%) and hemosiderin (29.85%) (Table 4) (Figures 1 and 2).

**Table 4:** Histopathological features of the vocal fold nodules and polyps stained with HE, Gomori trichrome and PAS.

Characteristics	Nodule		Polyp		Significance
	N	%	N	%	*p*
Epithelial					

Hyperplasia	46	82.14	34	50.75	0
Atrophy	7	12.5	21	31.34	0.013
Erosion	3	5.36	6	8.96	0.445
Dysplasia	0	0	0	0	1
Keratinization ortho	2	3.57	8	11.94	0.091
Keratinization para	19	33.93	7	10.45	0.001
Keratinization ortho/para	2	3.57	2	2.99	0.855

*Lamina propria*					

Edema	40	71.43	66	98.51	0
Fibrosis	32	57.14	11	16.42	0
Inflammatory infiltration	28	50	37	55.22	0.563

Basement membrane					

Thickened BM	56	100	0	0	0
BM thin/intact	0	0	67	100	0
BM focal/thickened	7	12.5	21	31.34	0

Vascular characteristics					

AMD	5	8.93	49	73.13	0
Mild angiectasia	45	80.36	14	20.9	0
Severe angiectasia	1	1.79	51	76.12	0
Recent hemorrhage	10	17.86	51	76.12	0
Hemosiderin	0	0	20	29.85	0

## DISCUSSION

Phonotrauma is largely responsible for the formation of the benign laryngeal lesions more frequently seen in daily practice^5^, ^6^. The clinical diagnosis of these lesions is usually difficult, generating many questions vis-à-vis the ultimate ENT diagnosis and speech therapy treatment. Usually, the lesions unresponsive to speech therapy are surgically removed and sent to pathology to define the type of lesion, with the aim of reaching the proper diagnosis. However, even from the standpoint of pathology, the differentiation of these lesions, especially among nodules and polyps, does not always occur, and it is often misdiagnosed as a nonspecific inflammatory process.

Nodules and polyps are the most common laryngeal lesions resulting from phonotrauma, and the distinction between them is often difficult, both macroscopically and microscopically. There is no defined histology pattern for these lesions. Thus, there is extensive research on the otorhinolaryngological and pathological correlation between nodules and polyps being published^7^, ^8^, ^9^.

From this observation, we led this study and found it possible to make the histological differentiation between nodules and polyps, through routine staining, contrary to the literature that suggests that histological parameters used to define laryngeal nodules and polyps are not well defined^10^, ^11^.

We first surveyed the incidence of these lesions in relation to gender. The literature shows a higher incidence of polyps in males and nodules in females, which was also observed in our study (64% of polyps in males and 86% of nodules in females)^12^. Only two studies that evaluated only vocal fold polyps, reported an increased occurrence of this type of lesion in females when compared to males, in contrast with our study^13^.

In our study we found a high correlation between the ENT clinical and pathological diagnoses (93.18%), in agreement with the literature^14^, with 91.5% of agreement. Only nine specimens (6.82%) had discordant diagnosis. According to the authors, the pathology differential diagnosis between nodules and polyps is the most difficult to perform in laryngeal biopsies and therefore must be made by means of an interactive relationship between the clinician and the pathologist. We believe that the high correlation between the ENT diagnosis and the pathologic features found in our study were possible because there is a greater integration between the otolaryngologist, pathologist and speech therapist, allowing them to use common terminology for these lesions.

Studies on the histological architecture of the vocal folds started from the description made by Hirano (1981)^15^ concerning the model called body-coverage. Since then, the knowledge of these layers, particularly the epithelium, *lamina propria* and basement membrane zone has become of paramount importance in the understanding the vocal mechanism. The analysis by conventional microscopy using routine stains such as hematoxylin and eosin can provide information to differentiate the lesions, although no specific feature is isolated from each lesion. Many authors believe that only routine staining with hematoxylin and eosin (HE) is not enough for the histological analysis of these lesions^16^, ^17^. Some authors have reported the importance of using other means such as electron microscopy and immunohistochemistry to differentiate these lesions^18^. In our studies we noticed that the use of other types of stains was important as PAS and Masson's trichrome: these histochemical stains are easily accessible in daily clinical pathology. We agree that the use of other methods provides for a more thorough evaluation, but are not easily accessible in everyday practice.

The aim of our study was to use routine staining methods in an attempt to establish the characteristics that can differentiate nodules and polyps in the daily practice, without resorting to special methods. Thus we investigated the histological features present in polyps and nodules using hematoxylin and eosin, Gomori trichrome and PAS for the histological analyses of the changes in epithelium, basement membrane, *lamina propria* and vascular aspects.

Regarding the histological features observed in vocal fold nodules and polyps, we found that the nodules had a predominance of epithelial changes, hyperplasia (82.14%) and parakeratosis keratinization (33.93%). As for the polyps, there was a predominance of atrophy (31.34%). Hyperplasia and parakeratosis was statistically significant and we considered it an important aspect of the histological differentiation between nodules and polyps, which differs from the study carried out by Lehrhoff and Rubin (1962)^19^ which considers the epithelial changes a factor of little relevance in the differentiation of such lesions. Whereas hyperplasia is the increase in cell number in response to a chronic trauma, its regression would be a return to the normal number of cells when the trauma ends, then we expect to have a regression of the edematous nodules (recent ones) with speech therapy.

By analyzing the *lamina propria*, we found a predominance of edema in polyps (98.51%) and fibrosis in the nodules (57.14%) which were considered significant characteristics (*p* = 0.001) to differentiate the lesions. Inflammatory infiltrate was found in both lesions, not being predominant in any of them. Some authors^20^, ^21^ have reported edema as a constant feature in vocal polyps and nodules, thus not serving as a differentiating factor. However, Kambic et al.^22^ reported the presence of sub-epithelial tissue edema, found a greater or lesser extent in laryngeal polyps.

Basement membrane analysis is considered one of the richest and most interesting parameters in the histological differentiation of vocal fold nodules and polyps. The literature is unanimous in pointing out the evidence of basement membrane duplication or thickening in vocal nodules^23^, ^24^. In this study, we found basement membrane thickening in nodules (100%) and it was thin/intact in polyps (100%). This feature is very important in the differentiation of the lesions, being significant in the pathological examination, also with a relationship between the change in basement membrane and voice use^25^. The nodules represent a response to repetitive traumas that cause a derangement/thickening to the basement membrane. Thus the clutter in the basement membrane of nodules is considered a typical response to vocal trauma^26^.

Within the vascular-changes parameter, mild angiectasia was predominant in nodules (80.36%). Polyps, however, have a deposition of amorphous material (73.13%), vascular clusters with marked angiectasia (76.12%), recent hemorrhage (76.12%) and hemosiderin (29.85%) (*p* < 0.005). These results show that the vascular aspects are an important parameter in the histopathological analysis and agree with previous studies^27^, ^28^. The vessel increase in polyps can be explained by the major impact trauma causes to these injuries, leading to recent hemorrhage, thrombosis, and capillary proliferation^29^. We wonder whether this would be one of the reasons for the persistence of polyps in speech therapy.

The speech traumas responsible for vocal nodules reach more superficial layers and less frequently the vessels of the submucosa, because vocal fold coverage moves independently of its body. The fact that recent nodules are more superficial and do not reach the submucosal vessels could justify the good response of these lesions in speech therapy, differently from what happens to polyps.

We have demonstrated that histological analysis by routine staining, through conventional microscopy, can provide important information. Thus, the combination of the histological traits observed in this study contributes to the differential diagnosis between vocal nodules and polyps. Hopefully, studies with specific concentration on molecular components can enhance our knowledge of these lesions, and the reason why they predominate in one gender and not the other, and perhaps explain the response of the lesion to speech therapy providing new surgical and speech therapy approaches.

## CONCLUSION

There was a high correlation between the ENT diagnosis and pathological diagnosis of vocal nodules and polyps. Nodules showed histopathological changes, predominantly epithelial alterations with hyperplasia and parakeratosis keratinization, *lamina propria* fibrosis, basement membrane thickening and mild angiectasia. However, polyps showed changes, predominantly *lamina propria* edema and vascular aspects, such as amorphous material deposits, marked angiectasia, recent hemorrhage and hemosiderin.
